# Evaporation of Methylammonium Iodide in Thermal Deposition of MAPbI_3_

**DOI:** 10.3390/nano11102532

**Published:** 2021-09-28

**Authors:** Ke Wang, Benjamin Ecker, Jinsong Huang, Yongli Gao

**Affiliations:** 1 Department of Physics and Astronomy, University of Rochester, Rochester, NY 14627, USA; kwang41@ur.rochester.edu (K.W.); becker@ur.rochester.edu (B.E.); 2 Department of Applied Physical Sciences, University of North Carolina at Chapel Hill, Chapel Hill, NC 27599, USA; jhuang@unc.edu

**Keywords:** methylammonium iodide, thermal evaporation, residual gas analyzer, X-ray photoelectron spectroscopy, morphology

## Abstract

Thermal evaporation is an important technique for fabricating methylammonium lead iodide (MAPbI_3_), but the process is complicated by the need to co-evaporate methylammonium iodide (MAI) and PbI_2_. In this work, the effect of water vapor during the thermal deposition of MAPbI_3_ was investigated under high vacuum. The evaporation process was monitored with a residual gas analyzer (RGA), and the film quality was examined with X-ray photoelectron spectroscopy (XPS). The investigations showed that during evaporation, MAI decomposed while PbI_2_ evaporated as a whole compound. It was found that the residual water vapor reacted with one of the MAI-dissociated products. The higher iodine ratio suggests that the real MAI flux was higher than the reading from the QCM. The XPS analysis demonstrated that the residual water vapor may alter the elemental ratios of C, N, and I in thermally deposited MAPbI_3_. Morphologic properties were investigated with atomic force microscopy (AFM), scanning electron microscopy (SEM), and X-ray diffraction (XRD). It was observed that a sample grown with high water vapor pressure had a roughened surface and poor film quality. Therefore, an evaporation environment with water vapor pressure below 10^−8^ Torr is needed to fabricate high quality perovskite films.

## 1. Introduction

Organometal halide perovskites have received much attention in recent years as an efficient light-absorbing material for use in solar cells, and device efficiency was able to reach 25.5% in 2020 [1,2,3]. This high efficiency is attributed to its outstanding optoelectronic properties, including its long diffusion length, long carrier lifetime, tunable bandgap, and high absorption coefficient [4,5,6,7,8]. Compared to traditional silicon solar cells, perovskite thin films can readily be made in a lab environment with techniques such as spin coating, blade coating, and thermal deposition. Thermal deposition is a mature technology that has been widely adopted in the coating and semiconductor industries, and it has demonstrated the ability to grow highly uniform perovskite thin films, often with fewer defects and ideal chemical stoichiometry [9,10,11,12]. The unique advantages of vacuum deposition include the controlled thickness, intrinsic purity of sublimed materials, easy preparation of multi-layer architectures for optimizing elementary processes (such as charge injection, transport, and recombination), and formation of desirable device structures such as tandem cells. Another advantage of thermal deposition is its low substrate-fabrication temperature, which is important for compatibility with plastic electronics, lightweight flexible devices, and combinations with traditional inorganic solar cells as tandem devices [13]. Furthermore, thermally evaporated thin films can be prepared in situ for surface-sensitive analytical investigations of the fundamental material properties of perovskites in regard to compatible surface quality [10,12,14]. More recently, it was proven that the thermal deposition of MAPbI_3_ is highly scalable in fabricating solar cells and mini-modules (21 cm^2^ active area); a remarkable record of 18.13% efficiency was achieved [15].

While the thermal deposition of perovskite has distinct advantages, it also has unique, unsolved issues. Olthof et al. found that that there is an induction period during which volatile compounds are formed, and the thickness of the induction layer could be 20–30 nm depending on the substrate material [16]. Bækbo et al. observed that MAI decomposed into CH_3_NH_2_ and HI, and the sticking coefficient of MAI was low because recombination from the decomposed species was necessary [17]. They also observed that the growth of MAI was relatively insensitive to the orientation and location of the substrate with respect to the evaporation source, and changing evaporation pressure was a better way to control the deposition rate. These results showed that the evaporation process of perovskite thin films is complicated. A better understanding of the process, especially of the dynamics during evaporation, is necessary to fully realize the photovoltaic and other potential applications of the perovskites. Our previous investigation [10] of the evaporated films revealed that the perovskite structure could be damaged and decomposed into a hydrocarbon complex, PbI_2_, gaseous NH_3_, and HI in the presence of water vapor. However, the interactions of the perovskite precursors (PbI_2_ and MAI) with residual gases while in the vapor phase during their depositions are poorly understood.

Here, we present our investigations of the dynamic process of the thermal deposition of MAPbI_3_. The partial vapor pressure changes of the perovskite precursors in the vapor phase were monitored with a residual gas analyzer (RGA), the evaporation rate of MAI was recorded with a quartz crystal microbalance (QCM), and the elemental ratio of the sample was characterized with X-ray photoelectron spectroscopy (XPS). We found that the residual water vapor inside the vacuum chamber interfered with the evaporation. The water pressure was reduced during MAI evaporation, indicating that a chemical reaction was taking place. As such, we propose a model, supported by partial pressure analysis and thermodynamics calculation, to explain this phenomenon. We also found that the reaction affected the composition and electronic structure of the perovskite thin films. We further observed that the MAI flux was significantly underestimated by the QCM. Additionally, atomic force microscope (AFM), scanning electron microscope (SEM), and X-ray diffraction (XRD) measurements showed that the film evaporated under high water vapor pressure had a small grain size, more voids, and excess PbI_2_, i.e., a generally rougher surface with poor film quality. The results suggest that controlling the water pressure and MAI evaporation rate are desirable for the thermal deposition of MAPbI_3_.

## 2. Experimental Section

PbI_2_ powder was purchased from Sigma-Aldrich Corporation, St. Louis, MO, USA (99% purity). The MAI powder was synthesized and provided by the collaborative group [18], MAI was synthesized from methylamine (40 wt.% in H_2_O; Sigma-Aldrich, and hydroiodic acid (57 wt.% in H_2_O (99.95%) with stabilizer; Sigma-Aldrich, St. Louis, MO, USA), as reported by Lee et al. [19]. The impurities of MAI play an important role in the fabrication process and have a significant impact on the performance of perovskite solar cells [20] Therefore, ensuring the purity of MAI is essential to ensure the quality of fabricated perovskite films. We examined three different MAIs prepared by our collaborator, Sigma-Aldrich, and Shanghai Zhenpin limited Company, Shanghai, China. The results are shown in Figure S1 and Table S1. We chose to use the MAI prepared by our collaborator because it had the most ideal ratio and almost no discernable impurity. The MAI and PbI_2_ precursors were loaded in tantalum boats, with a metal shield placed between them to avoid crosstalk. They were individually evaporated in a vacuum chamber that was initially continually pumped by a turbomolecular pump; later, we added an ion pump to reduce water vapor pressure. A thermal couple was attached to the center of each boat to monitor the evaporation temperature. The base pressure of the evaporation chamber was generally 1 × 10^−7^ Torr. An Extorr XT300(M) residual gas analyzer (Extorr Inc., New Kensington, PA, USA) was attached to the evaporation chamber to monitor partial vapor pressures in the chamber. A distilled water tube with a leak valve was also attached to the evaporation chamber to manually control the water vapor pressure. Two quartz crystal microbalances were attached slightly below the actual sample position to monitor the deposition rate and thickness of PbI_2_ and MAI. The thickness of the films was monitored with a quartz crystal microbalance (QCM), and the composition and crystal structure were examined by XPS and XRD.

Both PbI_2_ and MAI were degassed at an 80% power setting, and the temperature was kept near the evaporation point for 60 min. The MAI evaporation rate was set to 1 Å/min at 120 °C, while the PbI_2_ evaporation rate was set at 1 Å/min at 360 °C. The evaporation for each precursor lasted about 30 min, and the entire heating, evaporation, and cooling process comprised about 100 min. The partial vapor pressures were monitored and recorded with the RGA.

XPS measurements were performed in an ultra-high vacuum (UHV) analytical chamber with a base pressure of 8 × 10^−11^ Torr and high resolution XPS with a monochromatic Al Kα source (1486.6 eV). The X-ray gun ran at 10 kV and 20 mA, and the spot size on the sample surface was 0.6 mm in diameter. The typical probing depth of XPS is about 6 nm, which was much smaller than the thickness of the evaporated perovskite film, so our XPS could only measure the surface of the perovskite film. However, the consistency of the XPS and XRD results of the evaporated MAPbI_3_ films was examined and confirmed in our previous studies [10,12,14]. The spectra for the I 3d_5/2_, Pb 4f_7/2_, C 1s, and N 1s were collected to estimate the elemental ratio of the sample surface. The core level peaks were fitted with CasaXPS version 2.3. The Shirley-type correction was used to remove the secondary electron background, and the ratios of the Lorentzian and Gaussian line shapes were not fixed during the peak-fitting procedure. The elemental ratio of the sample surface was calculated by dividing the areas of the fitted curves by their atomic sensitivity factors. The samples were transferred from the evaporation chamber to the XPS analytical chamber with a detachable vacuum-sealed transfer arm.

The surface morphology was investigated with an NTMDT AFM microscope and a Zeiss Auriga SEM. The crystalline structure of the evaporated films was obtained with Bruker D8 Advance XRD System. The XRD measurements were performed under Cu Kα X-ray radiation operating at 40 kV and 30 mA using a step size of 0.03° and a time per step of 1 s. The experimental fitting of the X-ray data was carried out from 10 to 70° 2θ at a fixed omega angle of 1 degree.

## 3. Results and Discussion

### 3.1. MAI Dissociation in Thermal Deposition

**Figure 1:**
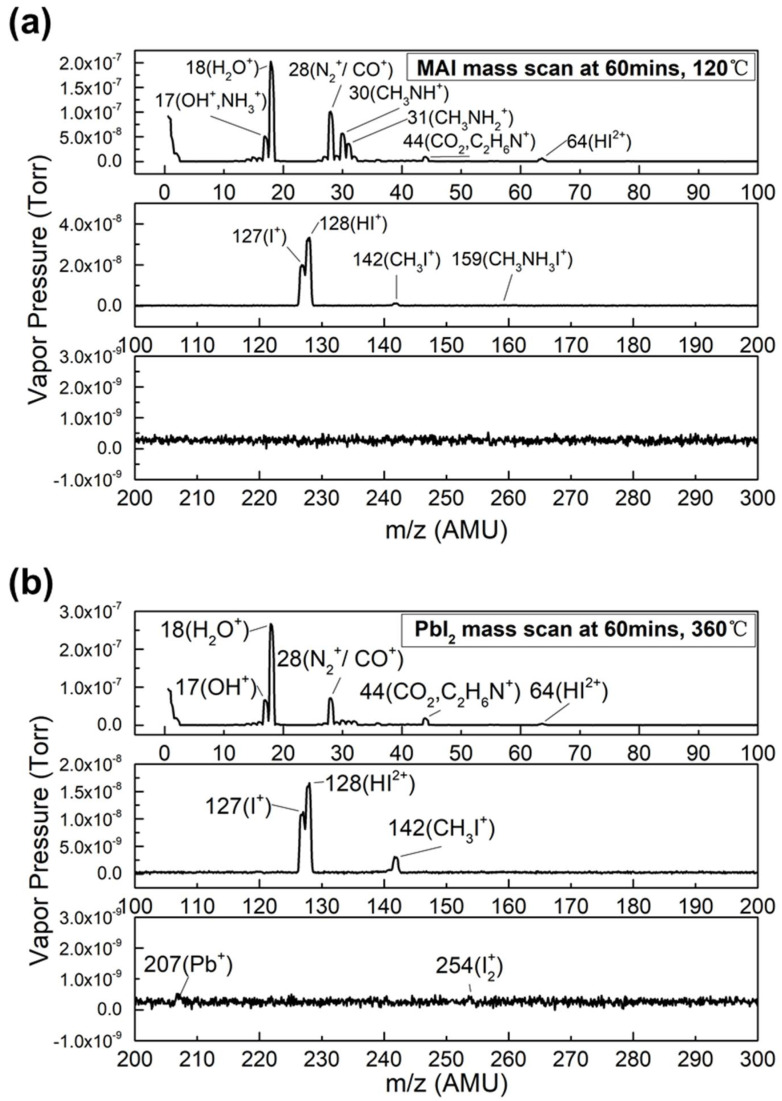
(**a**) Mass scan for MAI evaporated at 120 °C, showing the *m*/*z* from 0 to 300. (**b**) Mass scan for PbI2 evaporated at 360 °C, showing the *m*/*z* from 0 to 300.

In Figure 1, the mass scans are presented from 0 to 300 AMU for (a) MAI boat temperature of 120 °C and (b) PbI_2_ boat temperature of 360 °C. Figure 1a shows that water and water fragment (AMU 17) (with pressures of 2.02 × 10^−7^ and 5.10 × 10^−8^ Torr, respectively) were the dominant peaks in the spectrum. The other noticeable peaks are the MAI-dissociated products CO, CH_3_NH_2_, CH_3_NH, HI, and I. No MAI peak (AMU 159) was detected by RGA. In Figure 1b, the pressures of water and OH^+^ are both higher than that of MAI evaporation because of the higher evaporation temperature of PbI_2_. Additionally, the pressures of all MAI-dissociated products, except for CH_3_I, were greatly reduced. This suggests that even without MAI evaporation, MAI-dissociated products existed in the chamber. For both evaporations, Pb^+^ and I_2_^+^ were not detected by RGA. Table 1 shows the most noticeable peaks along with the most likely compounds that they are associated with. During the MAI evaporation, the main MAI peak was not detected by RGA, but the peaks of the MAI-dissociated products CO, CH_3_NH_2_, CH_3_NH, HI, and I were noticeable. This indicates that MAI did not evaporate as a whole molecule; instead, it thermally decomposed into its components in the vacuum during the evaporation. These results agree well with the observations of Bækbo, et al. [17]. For PbI_2_ evaporation, Pb (AMU 207) and I_2_ (AMU 254) peaks were not detected (as seen in Figure 1b), which suggests that PbI_2_ evaporates without dissociation. I, HI, and CH_3_I were the residual compounds from MAI evaporation.

**Figure 2:**
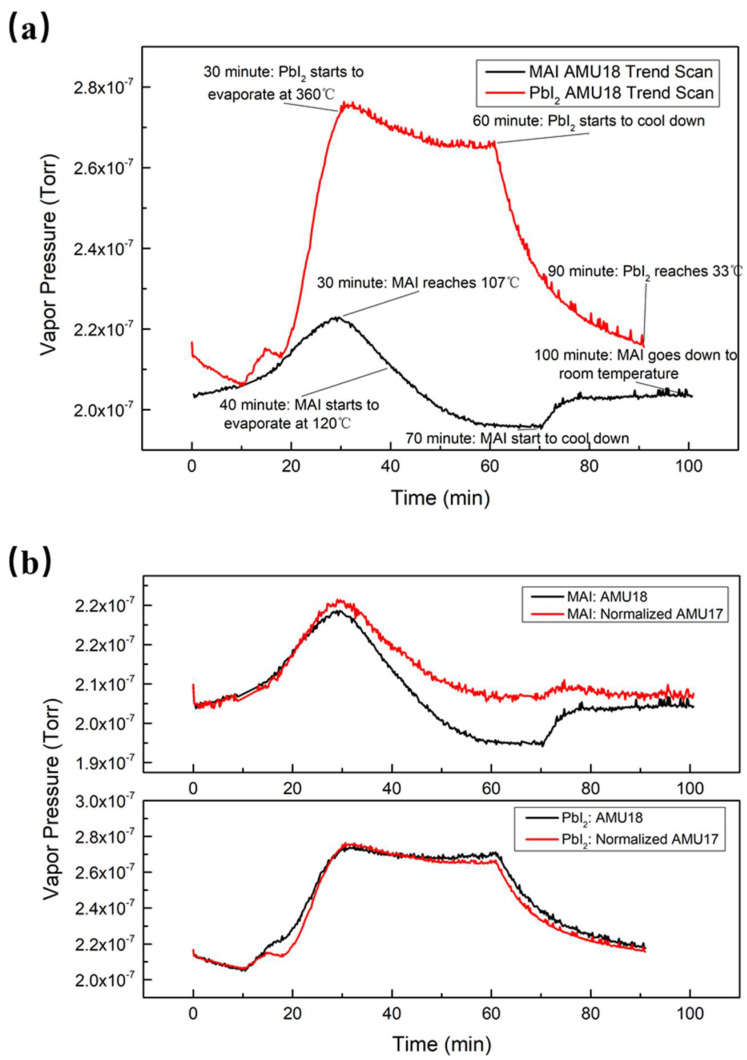
(**a**) Comparison of water (AMU 18) trend scans in MAI and PbI_2_ evaporation. (**b**) Comparison of water (AMU 18) and normalized AMU 17 trend scans in MAI and PbI_2_ evaporation.

The dynamics of the evaporation were monitored by observing the time evolution of the RGA scans. In Figure 2a, a typical evolution pattern of PbI_2_ evaporation is shown. The partial pressure of water initially increased as the temperature rose. Once the temperature was stabilized, the water pressure slightly decreased as the new equilibrium state was established. When the evaporation was finished, the water pressure returned to its initial pressure. The partial pressures of water vapor behaved differently in MAI and in PbI_2_ evaporation, as shown in Figure 2a. For MAI evaporation, the water vapor pressure increased from the beginning of heating to about 30 min due to the heating of both the MAI boat and its surrounding area that desorbed the water molecules from the boat and the inner chamber wall. As the evaporation progressed, the water pressure decreased from its peak value of 2.20 × 10^−7^ Torr at 30 min to below the base value of 2.02 × 10^−7^ Torr after 47 min, indicating the occurrence of a chemical process that consumed water during the evaporation.

The water pressure behavior during the PbI_2_ evaporation was qualitatively different from that during MAI evaporation. The water pressure increased in the initial stage of evaporation, reaching a maximum value of 2.75 × 10^−7^ Torr at 30 min, similar to that of MAI. The maximum water vapor pressure was higher than that in MAI evaporation because the evaporation temperature of PbI_2_ is much higher than that of MAI. The pressure stabilizes after 50 min at 2.67 × 10^−7^ Torr, far above the baseline of water and in sharp contrast to the case of MAI. The reduction of water during evaporation may have been caused by the absorption of water by MAI on the inner chamber wall and reabsorption by the inner chamber wall. After turning off the evaporation power supply, both temperatures quickly decreased and the water vapor pressures returned to their initial values.

As indicated in Figure 1 and Table 1, AMU 17 could be both NH_3_^+^ and OH^+^ as water fragment. We therefore compared the H_2_O^+^ peak at AMU 18 to that at AMU 17 to see if there was any NH_3_^+^ contribution. Figure 2b shows a comparison of water and normalized AMU 17 trend scans in both MAI and PbI_2_ evaporation. The two trend scans can be seen to practically overlap for PbI_2_ evaporation, while the water trend was further decreased than that of AMU 17 for MAI. The residual gas molecules were electron impact-ionized, and then the quadrupole separated all the ions so that we could measure their mass-to-charge ratios and current. These ions could have been molecular fragments or from a mixture of molecules: this is the cracking pattern of each molecule. The ionization energy of our RGA was 70 eV; under this configuration, we observed approximately 25% water molecules cracking into OH^−^ [21]. However, NH_3_ has the same molecule weight of 17, which is the same as OH^−^. To further investigate AMU 17’s composition, we normalized AMU 17’s trend scan to reach the same initial value as that of the water in Figure 2b. Water and AMU 17’s curves were almost coincident in PbI_2_ evaporation, which suggests that most of AMU 17 was OH^−^. However, AMU 17 did not decrease as much as water in MAI evaporation, which indicates that AMU 17 could be a mixture of OH^−^ and NH_3_.

### 3.2. MAI Dissociation Mechanism

**Figure 3:**
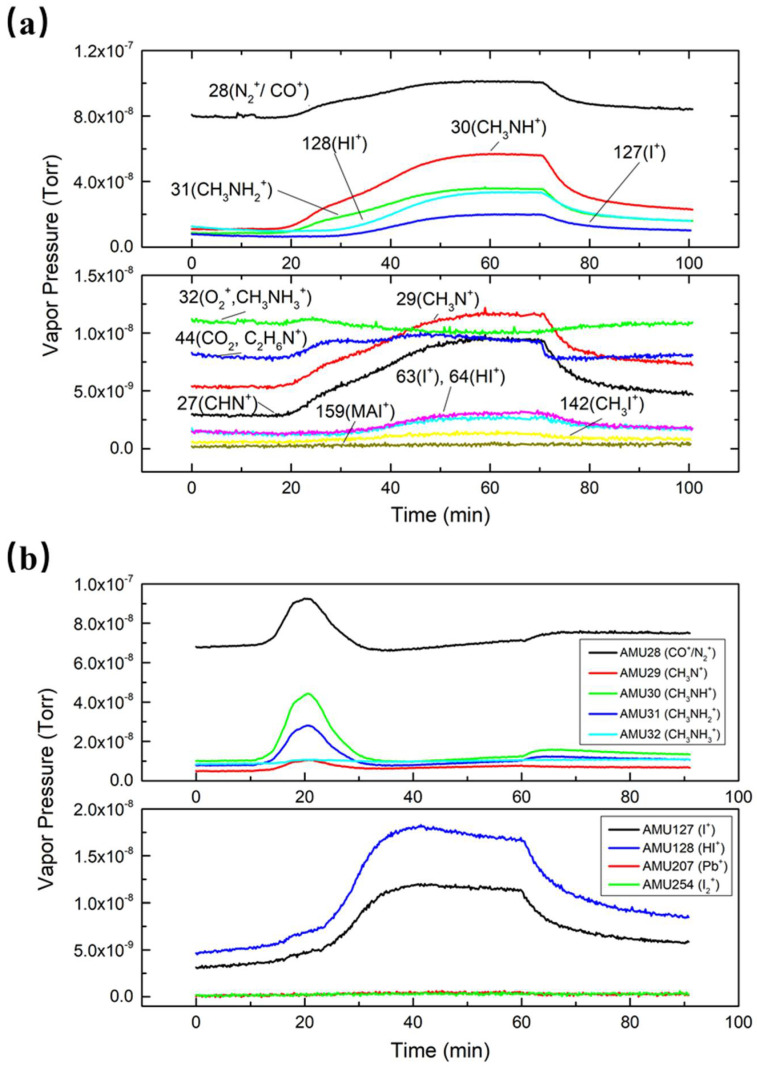
(**a**) Trend scans for MAI-dissociated products evaporated at 120 °C. (**b**) Trend scans for MAI^−^ and PbI_2_-dissociated products in PbI_2_ evaporation.

All residual gases were monitored and recorded with RGA. The trend scans of MAI-dissociated products are shown in Figure 3a, which illustrates the partial pressure changes for the whole heating–evaporation–cooling process. We first degassed the RGA filament and then started evaporation at 10 min when the detected pressures were stabilized. It took about 30 min to reach the evaporation temperature. As can be seen in the figure, the vapor pressures of MAI-dissociated products rose with the increase in the temperature while the MAI curve stayed flat, which means there was no MAI detected by RGA. These results support the notion that MAI does not evaporate as a whole compound but instead dissociates into fragments, as observed by Bækbo et al. [17].

In order to compare partial pressure changes for PbI_2_ and MAI, we also collected data for each individual compound in PbI_2_ evaporation. Figure 3b shows that there were no increases for both Pb (AMU 207) and I_2_ (AMU 254) during the PbI_2_ evaporation process, which further proves that PbI_2_ evaporates as a whole compound. However, due to the limitations of the RGA, PbI_2_ molecules were out of the detecting range. I and HI were suspended and accumulated in the evaporation chamber after each MAI evaporation. Once the temperature increased, I and HI left the inner chamber wall, thus explaining the strong presence of the two compounds. Meanwhile, at the early stage of PbI_2_ evaporation, several MAI-dissociated products were also detected, even though MAI was not evaporated. This further confirms that these dissociated products were floating in the chamber and sticking to the PbI_2_ boat and nearby areas.

**Figure 4:**
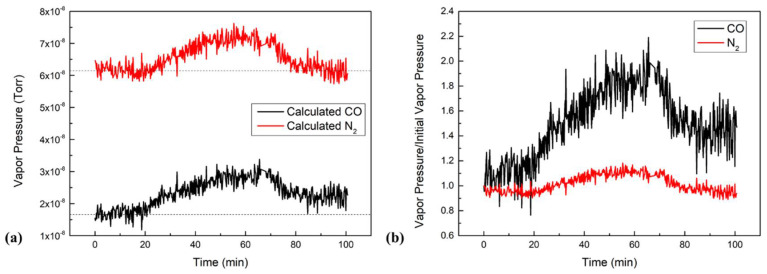
(**a**) Comparison of calculated CO and N_2_ trend scans in MAI evaporation. (**b**) Comparison of the real vapor pressure and initial pressure of CO and N_2_.

Among all the MAI-dissociated products, AMU 28 increased the most. However, it was hard to identify whether CO or N_2_ was produced because their trend scan to reach the same initial value as that peaks coincided with each other. To further investigate the composition of AMU 28, we used the cracking patterns from the RGA manual provided by the manufacturer to calculate the partial pressures for each component. AMU 28 was found to consist of three parts: N2, CO, and CO_2_ fragments. We found ~11.4% of AMU 44 that was actually in AMU 28. AMU 12 was found to represent ~4.5% of CO. Therefore, the pressure of N_2_ could be obtained by subtracting CO and CO_2_ fragments from total pressure of AMU 28. Following this calculation, the trend scans for both CO and N_2_ shown in Figure 4a were obtained. Both curves increased during the heating stage then stabilized when the boats reached evaporation temperatures, and they both decreased after turning off the power supplies for the MAI boat. However, N_2_′s final pressure was 6.16 × 10^−8^ Torr (which was similar to its initial pressure), while CO’s final pressure was 2.30 × 10^−8^ Torr (which was higher than its initial pressure of 1.69 × 10^−8^ Torr). The increase of N_2_ pressure was due to the heating effect of the evaporation boat and its surroundings, and it returned to its equilibrium position when the temperature again decreased. To have a more comparable figure, we plotted Figure 4b by dividing each vapor pressure by its initial pressure. Then, we obtained the ratio of the on-going vapor pressure and the initial pressure. The figure shows that CO pressure increased by about 90% during the evaporation, while N_2_ only increased by 10%. After evaporation, CO still increased by about 50% and N_2_ fell back to its initial pressure. Therefore, the total pressure of N_2_ did not increase, indicating that N_2_ was not produced during evaporation. On the contrary, the increase in total CO pressure suggests that CO was produced during the evaporation.

The data of Figure 4 show that the partial pressures of CO and N_2_ increased by 1.23 × 10^−8^ and 1.14 × 10^−8^ Torr, respectively. Since N_2_ did not participate in the reaction, the increase was due to the heating effect that occurred as it was degassed from MAI during the heating process, which means that it increased 19% from its initial pressure. We assumed that the heating effect also applied to CO and NH_3_, so we were able to deduce that the increases of 3.20 × 10^−9^ and 4.10 × 10^−10^ Torr of CO and NH_3_, respectively, were due to heating, which were the 19% of their initial pressure. Therefore, the net increases of the CO and NH_3_ due to the water reaction were 9.10 × 10^−9^ and 1.94 × 10^−9^ Torr, respectively.

Based on this discussion, we propose the following reaction for water during MAI evaporation:

CH_3_NH_2_ + H_2_O(g)→CO(g) + NH_3_(g) + 2H_2_(g)

In order to understand whether this reaction is thermodynamically spontaneous, its Gibbs free energy of formation Δ_f_*G* needed to be calculated. The standard Gibbs free energy of formation Δ_f_*G^o^* was found to be 51.06 kJ/mol, which means the reaction is not thermodynamically spontaneous in the standard state. However, when using the states from the evaporation period (including pressure and temperature for each reactant and product), Δ_f_*G* was found to be negative. In this case, we used the pressures of CO, NH_3_, H_2_, CH_3_NH_2_, and H_2_O at 120 °C for 60 min. Among the five chemical substances, the pressures of CH_3_NH_2_ and H_2_ were 3.57 × 10^−9^ and 1.68 × 10^−9^ Torr, respectively, as directly obtained by RGA. Those for H_2_O, CO, and NH_3_ were 2.61 × 10^−8^, 2.92 × 10^−8^, and 4.50 × 10^−9^ Torr, respectively. These values were calculated based on the cracking patterns and the results discussed earlier [21]. R is ideal gas constant and T is 393.15 K, plugging these values into the Equation (1) revealed that Δ_f_*G* for this reaction is −124.65 kJ/mol under the evaporation conditions, thus implying that the reaction is thermodynamically spontaneous. The reaction may occur on the sample surface during evaporation.

(1) $$\Delta_{f}G=\Delta_{f}G^{o}+\mathrm{RT}\ln\left(\frac{P_{CO}\times P_{{NH}_{3}}\times P_{H_{2}}^{2}}{P_{{CH}_{3}{NH}_{2}}\times P_{H_{2}O}}\right)$$

Our observations and discussion suggest that when we evaporated MAI, it decomposed into CH_3_NH_2_, HI, and other dissociated products. Thus, water vapor could have collided and reacted with CH_3_NH_2_ when it was dissociated from MAI. The consumption of CH_3_NH_2_ also reduced the amount of CH_3_NH_2_ that reached the sample surface, which led to an imbalance of the CH_3_NH_2_, HI, and PbI_2_ ratio and resulted in an excess of PbI_2_. As such, water vapor may have reduced the conversion rate of perovskite precursors and the elemental ratio of the sample surface. In this case, to grow perovskite film on the sample surface, both CH_3_NH_2_ and HI needed to be present at the same time as PbI_2_ to form perovskite.

### 3.3. Effects from Different Evaporation Conditions

We evaporated a sample under a water vapor pressure of 1.0 × 10^−6^ Torr and an MAI temperature of 139 °C (HW/HT), and then we used XPS to see whether the chemical composition was different from the sample grown at 2.08 × 10^−9^ Torr of water vapor pressure and 139 °C of MAI temperature (LW/HT). First, we evaporated two 200 Å thick perovskite film on a highly oriented pyrolytic graphite (HOPG) substrate under water vapor pressures of 1.0 × 10^−6^ and 2.08 × 10^−9^ Torr. The high water vapor pressure was controlled by a leak valve that was connected to a distilled water tube. By leaking in distilled water, we manually set the water vapor pressure to 1.0 × 10^−6^ Torr, stabilized it for 20 min, and then started evaporation. The lower water vapor pressure was achieved with an ion pump. The evaporation conditions for the two samples was the same as those of precursor evaporation. After the evaporation, the samples were transferred to the analytical chamber in a vacuum-sealed transfer arm for XPS measurements.

**Figure 5:**
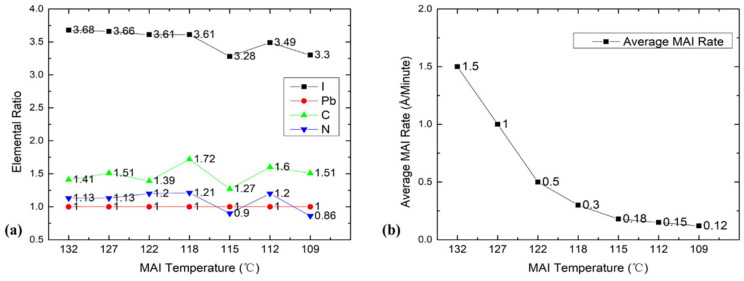
(**a**) Elemental ratios of the MAPbI_3_ films evaporated at different MAI evaporation temperatures and 1.75 × 10^−8^ Torr of water vapor pressure. (**b**) Average MAI rate from QCM reading as a function of MAI boat temperature.

To examine the effects of different MAI temperatures, we evaporated seven MAPbI_3_ films at different MAI evaporation temperatures from 132 to 109 °C at 1.75 × 10^−8^ Torr of water vapor pressure, and then we analyzed film composition with XPS. The results are shown in Figure 5. The MAI evaporation rates according to the QCM for these temperatures were 1.5, 1.0, 0.5, 0.3, 0.18, 0.15, and 0.12 Å/min. From 132 to 118 °C, the chemical composition of the film was quite uniform for all four elements, except a spike of C at 118 °C that was likely spurious because we did not see it in our many repeating measurements. Once we lowered the temperature to 109 °C (LW/LT), the iodine and nitrogen ratios were obviously lower than their initial ratios at 132 °C. It is worth noting that the ion pump consumed water and produced H_2_. The pressures of H_2_ before and after turning off the ion pump are displayed in Figure S2, which shows that H_2_ pressure was 2.50 × 10^−8^ Torr when the ion pump was on and quickly dropped to 1.30 × 10^−8^ Torr when the ion pump was turned off, which suggests that the ion pump produced H_2_ at 1.20 × 10^−8^ Torr. The pressures of related chemical substances can be seen in Figure S3. To check the effect of this portion of H_2_ on the thermodynamics of reaction, we calculated the Gibbs free energy of formation at 30 min with raw H_2_ pressures and the net residual H_2_ pressure; the results were −94.11 and −95.52 kJ/mol, respectively. These two values were similar and still thermodynamically favored the reaction. We found that the water vapor pressure first increased to 3.55 × 10^−8^ Torr at 132 °C and then slowly dropped to 2.38 × 10^−8^ Torr when the MAI temperature reached 109 °C. Though the MAI rate dropped from 1.5 to 0.12 Å/min, the iodine ratio only dropped to 3.3. Even QCM reading of MAI at 109 °C is 1/8 of PbI_2_ showed that the film stoichiometry remained OK. The drop in the elemental ratios was not as large as that of evaporation rate, which indicates that the MAI QCM reading underestimated the amount of MAI. Thus, we had an excess of MAI in the chamber, and the real MAI rate was much higher than that shown by the QCM reading; thus, 2 × 10^−8^ Torr of water pressure may not be enough to make a dramatic change with a much stronger MAI flow in terms of chemical composition. The drop of C, N, and I at 115 °C may be explained by the oversupply of MAI, as well as fluctuations in the choice of XPS measurement spot. (More LW/LT ratios can be found in Table S2.) In this case, high water pressure could have made a considerable difference with a lower MAI evaporation temperature. Kim et al. also reported similar QCM reading underestimations of MAI flux. They explained that due to the poor adhesion of MAI on the surface of the sensor crystals, the deposition rate of MAI could be increased in the presence of PbI_2_ [22]. In order to investigate how water pressure affected the evaporation at a low MAI temperature, we increased water pressure to 1.0 × 10^−6^ Torr and then measured sample growth at an MAI temperature of 109 °C (HW/LT). The RGA spectra are shown in Figure S4. The calculated pressures at CO, NH_3_, H_2_, CH_3_NH_2_, and H_2_O at 109 °C for 150 min were 9.60 × 10^−8^, 3.40 × 10^−8^, 2.03 × 10^−8^, 1.89 × 10^−8^, and 1.16 × 10^−7^ Torr, respectively. The Gibbs free energy for this condition was −108.38 kJ/mol, which still supports our previous assumption.

### 3.4. Characterization of MAPbI_3_ Films

**Figure 6:**
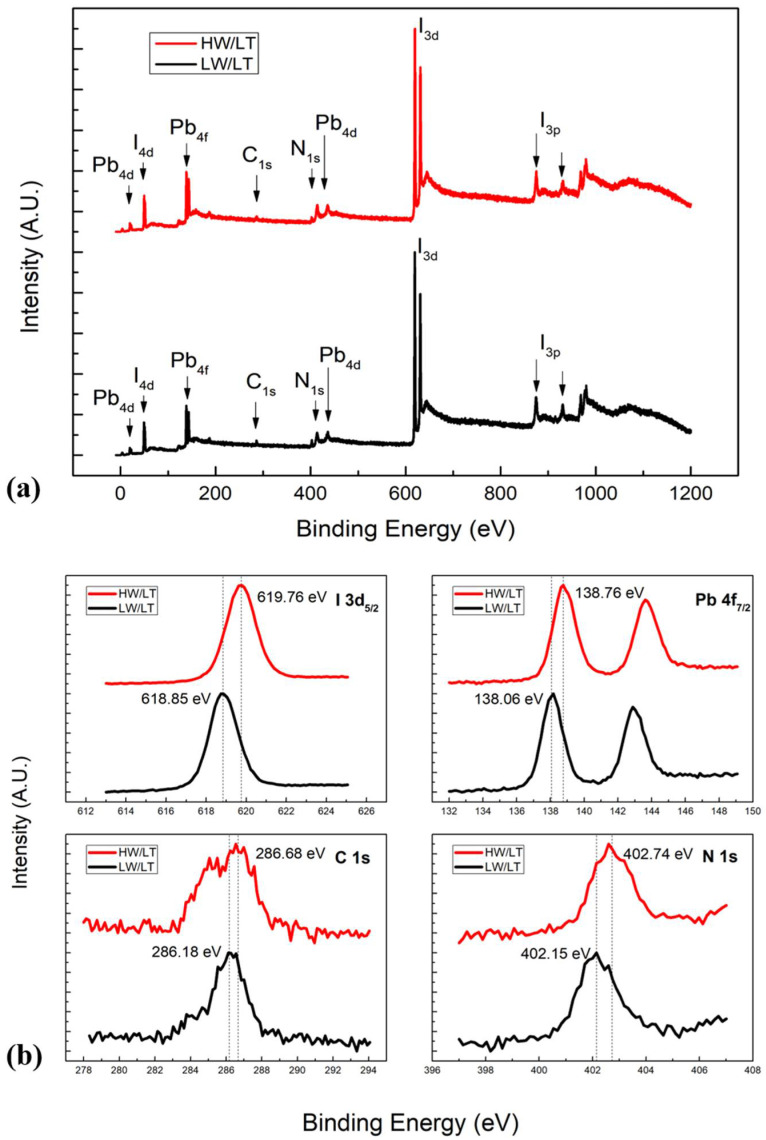
(**a**) XPS full scan comparison of HW/LT and LW/LT films. (**b**) I 3d_5/2_, Pb 4f_7/2_, C 1s, and N 1s core level spectra comparison of HW/LT and LW/LT films.

An elemental ratio comparison of LW/LT and HW/LT samples is shown in Table 2. It is clear that we had an overall ratio reduction for every element, which is different from the results shown in Figure 5. In the presence of high water pressure and an MAI temperate of 109 °C, perovskite carbon (286 eV) decreased by 46.4%, nitrogen decreased by 27.9%, and iodine decreased by 13%. This result shows that water vapor strongly affects perovskite film formation with a low MAI flux. This can be explained by the fact that partial CH_3_NH_2_ and other MAI-dissociated products were consumed by water vapor during the evaporation, as well as by the fact that the reaction left excess HI in the chamber that caused iodine to reduce less. A comparison of XPS survey scans is shown in Figure 6, where XPS features can be seen to be similar to each other, indicating their similar chemical compositions. Since both samples were grown under the same conditions except for water vapor pressure, comparing the core level spectra allowed us to find that there was no significant change in the spectral shape but a rigid shift of about 0.59 eV to the higher binding energy (BE) in the HW/LT condition. The observed rigid shift was unlikely due to charging because MAPbI_3_ film is relatively thin (200 Å), the material is of relatively high conductivity [23], and both the HW/LT and LW/LT samples are of the same thickness. The results suggest that the presence of high water vapor pressure moved the Fermi level of the perovskite to the higher binding energy region by 0.59 eV, indicating that the Fermi level is much closer to the bottom of the conduction band. This was because water vapor reduced the amount of MAI in the HW/LT sample and made it rich in PbI_2_, resulting in the n-doping of the sample (as previously observed in PbI_2_-rich films) [4,9,12]. In that sense, water vapor acts as an n-dopant of MAPbI_3_.

Figure 3b shows that MAI-dissociated products were floating and accumulated in the chamber after each evaporation. They also enhanced the iodine-rich environment in the chamber, which may have led to an iodine surplus on the sample surface. Therefore, the existence of water vapor in the evaporation chamber could have caused the elemental ratio deficiency of the film. The excess iodine may have served as the interface recombination center, thus limiting the performance of the perovskite device [24]. The evaporated film may have become less stable in the ambient environment when a considerable amount of water vapor was present during evaporation, which may have caused the rapid degradation of the film. Accordingly, water vapor pressure should be kept as low as possible during evaporation. In order to obtain a well-controlled MAPbI_3_ thin film with good chemical stoichiometry, a water-free chamber environment is needed. An attached ion pump could reduce water vapor pressure to as low as 10^−9^ Torr region, as well as reducing the pressure of other residual chemical substances from previous MAI evaporation to keep the chamber environment relatively clean. Figures S5 and S6 show the chamber environment before HW and LW evaporations and the chamber setup. Those of AMU 18 and AMU 28 are the most noticeable peaks in the spectra. During HW evaporation, water was the dominate peak because it was manually injected into the chamber to increase the water vapor pressure. However, in LW evaporation, water was effectively reduced with an ion pump and AMU 28 became the dominate peak. The spectra show that there was no major MAI-dissociated products before the evaporations, indicating that our chamber provided a clean environment. In addition, Figure S7 shows that the majority of the AMU 32 in MAI evaporation was CH_3_NH_3_^+^. And it was consumed during the evaporation. The impact of the evaporation boat was also examined. We heated an empty boat to 120 °C and tracked the pressure of several compounds. The results are shown in Figure S8, indicating the pressure change was neglectable before and after heating.

**Figure 7:**
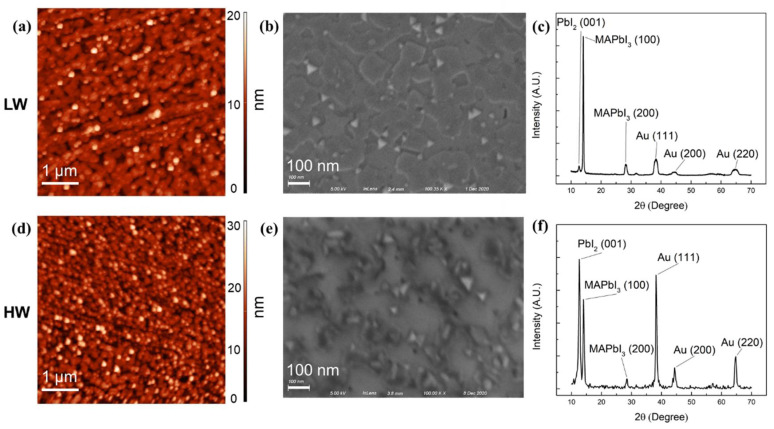
(**a**) AFM, (**b**) SEM, and (**c**) XRD images for the LW-evaporated thin film. (**d**) AFM, (**e**) SEM, and (**f**) XRD images.

We also performed AFM and SEM to investigate effects on sample morphology. AFM and SEM image comparisons of LW and HW samples are shown in Figure 7a,b,d,e. AFM measurements were performed at room temperature (RT) and 25.1% relative humidity (RH). The root mean square (RMS) of the LW-evaporated film was 2.08 nm, which suggests that film surface was extremely uniform. However, the RMS of the HW-evaporated film significantly increased to 13.78 nm. The other statistic parameters of AFM can be found in Table S3. SEM images further confirmed the surface uniformity difference between the LW and HW samples. The average grain sizes of the LW and HW samples were about 100 and 50 nm, respectively. The voids on the HW sample were much bigger than those on the LW ones. This indicates that water vapor played a destructive role in terms of surface uniformity during the evaporation. The grain size of perovskite plays an important role in device performance. Previous reports have shown that with the increasing grain size of perovskites, all photovoltaic parameters such as open-circuit voltage (Voc), short-circuit current density (Jsc), fill factor (FF), and power conversion efficiency (PCE) are improved. In addition, a large grain size could reduce bulk defects and pin holes at grain boundaries, thus enhancing the long term stability of a device [25,26,27,28]. Surface morphology also has a great impact on the optical properties. Droseros et al. found that photoluminescence (PL) is enhanced with grain size reductions of MAPbBr_3_ thin film [29]. Yan et al. reported that the grain size of MAPbBr_3_ thin film can be modified by changing the molar ratio of the organic to inorganic precursor, and PL emissions can reach their maximum when the molar ratio is two [30]. Falsini et al. showed that there are two PL emissions in CsPbCl_3_ thin film, and these emissions are attributed to the different morphologies of the crystallites [31].

Furthermore, the XRD spectra shown in Figure 7c,f demonstrate the crystallinity of the LW- and HW-evaporated films, respectively. The thin films were grown on an Au-coated Si wafer with a thickness of 800Å because the graphite substrate became uneven after multiple exfoliations, which made it unsuitable for XRD measurement because XRD requires larger film thicknesses. For both films, the perovskite, PbI_2_, and Au from the substrate can be identified in the spectra. Two perovskite peaks were observed in the LW sample at 14.07° and 28.31°, and these were assigned to the (100) and (200) lattice planes, respectively. The other three peaks at 38.29°, 44.32°, and 64.68° correspond to the (111), (200), and (220) diffractions of the Au substrate, respectively. The PbI_2_ peak located at 12.65° corresponded to the (001) lattice plane. For LW sample, it is obvious that perovskite peaks were the dominant features of the spectrum but PbI_2_ had a weak intensity, suggesting that the surface only had a small amount of PbI_2_. On the contrary, for the HW sample, the intensities of PbI_2_ and Au peaks greatly increased and even overtook the domination of perovskite peak. Based on our discussion, these can be explained by the idea that some MAI were consumed by water vapor during the evaporation, which led to excess PbI_2_ on the surface. Due to the crystallization of PbI_2_, voids may have been generated in the film and further exposed the Au substrate as a PbI_2_ crystal aggregate, thus leading to the increase of Au peak intensity.

## 4. Conclusions

In this study, we investigated the role of water vapor during the thermal evaporation of MAPbI_3_. We confirmed that MAI evaporates not as a whole compound but as CH_3_NH_2_, HI, and other dissociated products, while PbI_2_ evaporates as a whole compound. We also observed that CH_3_NH_2_ reacts with water vapor during evaporation. A chemical reaction for CH_3_NH_2_ and water vapor was proposed, as the Gibbs free energy of formation showed that the reaction is thermodynamically spontaneous under the evaporation condition. The elemental ratios of the evaporated films showed that MAI flux was underestimated by the QCM. This means the oversupplied MAI could lead to higher iodine ratios, which may limit device performance. When the MAI rate was reduced, the water vapor was able to significantly reduce the elemental ratios. This research indicates that water vapor is involved in the thermal deposition of MAPbI_3_ and has an impact on the deposition’s chemical stoichiometry. The XPS spectra showed that the peak positions had a rigid shift toward a higher BE region in HW/LT condition, which suggests that the water vapor acts as an n-dopant. Rougher surface and poorer film quality were confirmed with AFM, SEM, and XRD. We also found that reducing water vapor pressure is desirable for growing high-quality MAPbI_3_ film via thermal evaporation.

## Figures and Tables

**Table 1:** Assignments of parent and fragmentation peaks in the RGA spectra.

*m*/*z*	Compound	Likely Parent Molecule
12	C^+^	C, CO, and CO_2_ fragments
14	N^+^	N_2_ fragment
15	CH_3_^+^	CH_3_NH_2_ fragment
16	O^+^, CH_4_^+^	O_2_ fragment and CH_3_NH_2_ fragment
17	NH_3_^+^, OH^+^	CH_3_NH_2_fragment and H_2_O fragment
18	H_2_O^+^	H_2_O parent peak
27	CHN^+^	CH_3_NH_2_ fragment
28	CO^+^, N_2_^+^	CO parent peak, CO_2_ fragment, and N_2_ parent peak
29	CH_3_N^+^	CH_3_NH_2_ fragment
30	CH_3_NH^+^	CH_3_NH_2_ fragment
31	CH_3_NH_2_^+^	CH_3_NH_2_ fragment
32	O_2_^+^, CH_3_NH_3_^+^	O_2_ parent peak and CH_3_NH_3_I fragment
63	I^+^	HI fragment
64	HI^+^	HI fragment
127	I^+^	HI fragment
128	HI^+^	HI parent peak
142	CH_3_I^+^	CH_3_I parent peak
159	CH_3_NH_3_I^+^	CH_3_NH_3_I parent peak

**Table 2:** Elemental ratio comparison of LW/LT MAPbI_3_ and HW/LT MAPbI_3_.

Element	C (286 eV)	N	Pb	I
LW/LT	1.51	0.86	1	3.30
HW/LT	0.81	0.62	1	2.87

## Data Availability

The raw and processed data will be provided upon reasonable request.

## Supplementary Materials

The following are available online at https://www.mdpi.com/article/10.3390/nano11102532/s1, Figure S1: XPS spectra for currently used MAI, Figure S2: H_2_ pressure before and after turning off ion pump, Figure S3: RGA spectra for LW/LT sample, Figure S4: The RGA scans of major molecules in the HW/LT evaporation, Figure S5: RGA spectra of chamber environment before evaporations, Figure S6: Illustration of evaporation chamber, Figure S7: Comparison of AMU 16 and AMU 32 trend scans in MAI and PbI_2_ evaporation, Figure S8: RGA spectra of selected compounds of an empty boat at 120 °C., Table S1: The elemental ratio comparison of currently used MAI powder and two previous ones, Table S2: The elemental ratios of 10 LW/LT samples, Table S3: Comparison of AFM statistic parameters of LW and HW samples.

## References

[1] Snaith H.J. (2013). Perovskites: The Emergence of a New Era for Low-Cost, High-Efficiency Solar Cells. J. Phys. Chem. Lett..

[2] Liu D., Kelly T.L. (2013). Perovskite Solar Cells with a Planar Heterojunction Structure Prepared Using Room-Temperature Solution Processing Techniques. Nat. Photonics.

[3] Best Research-Cell Efficiency Chart (National Renewable Energy Laboratory). https://www.nrel.gov/pv/cell-efficiency.html.

[4] Xie H., Liu X., Lyu L., Niu D., Wang Q., Huang J., Gao Y. (2015). Effects of Precursor Ratios and Annealing on Electronic Structure and Surface Composition of CH3NH3PbI3 Perovskite Films. J. Phys. Chem. C.

[5] Kim H.-S., Lee C.-R., Im J.-H., Lee K.-B., Moehl T., Marchioro A., Moon S.-J., Humphry-Baker R., Yum J.-H., Moser J.E. (2012). Lead Iodide Perovskite Sensitized All-Solid-State Submicron Thin Film Mesoscopic Solar Cell with Efficiency Exceeding 9%. Sci. Rep..

[6] Dunlap-Shohl W.A., Younts R., Gautam B.R., Gundogdu K., Mitzi D.B. (2016). Effects of Cd Diffusion and Doping in High-Performance Perovskite Solar Cells Using CdS as Electron Transport Layer. J. Phys. Chem. C.

[7] Abrusci A., Stranks S.D., Docampo P., Yip H.-L., Jen A., Snaith H. (2013). High-Performance Perovskite-Polymer Hybrid Solar Cells via Electronic Coupling with Fullerene Monolayers. Nano Lett..

[8] Stranks S.D., Eperon G.E., Grancini G., Menelaou C., Alcocer M.J.P., Leijtens T., Herz L.M., Petrozza A., Snaith H.J. (2013). Electron-Hole Diffusion Lengths Exceeding 1 Micrometer in an Organometal Trihalide Perovskite Absorber. Science.

[9] Wang Q., Shao Y., Xie H., Lyu L., Liu X., Gao Y., Huang J. (2014). Qualifying Composition Dependent p and n Self-Doping in CH3NH3PbI3. Appl. Phys. Lett..

[10] Wang C., Li Y., Xu X., Wang C., Xie F., Gao Y. (2016). Degradation of Co-Evaporated Perovskite Thin Film in Air. Chem. Phys. Lett..

[11] Liu M., Johnston M., Snaith H. (2013). Snaith, Efficient Planar Heterojunction Perovskite Solar Cells by Vapour Deposition. Nature.

[12] Li Y., Xu X., Wang C., Wang C., Xie F., Yang J., Gao Y. (2015). Degradation by Exposure of Coevaporated CH_3_NH_3_PbI_3_ Thin Films. J. Phys. Chem. C.

[13] Ávila J., Momblona C., Boix P.P., Sessolo M., Bolink H.J. (2017). Vapor-Deposited Perovskites: The Route to High-Performance Solar Cell Production?. Joule.

[14] Li Y., Xu X., Wang C., Ecker B., Yang J., Huang J., Gao Y. (2017). Light-Induced Degradation of CH_3_NH_3_PbI_3_ Hybrid Perovskite Thin Film. J. Phys. Chem. C.

[15] Li J., Wang H., Chin X.Y., Dewi H.A., Vergeer K., Goh T.W., Lim J.W.M., Lew J.H., Loh K.P., Soci C. (2020). Highly efficient thermally co-evaporated perovskite solar cells and mini-modules. Joule.

[16] Olthof S., Meerholz K. (2017). Substrate-Dependent Electronic Structure and Film Formation of MAPbI3 Perovskites. Sci. Rep..

[17] Bækbo M.J., Hansen O., Chorkendorff I., Vesborg P.C.K. (2018). Deposition of Methylammonium Iodide via Evaporation—Combined Kinetic and Mass Spectrometric Study. RSC Adv..

[18] Bi C., Wang Q., Shao Y., Yuan Y., Xiao Z., Huang J. (2015). Non-Wetting Surface-Driven High-Aspect-Ratio Crystalline Grain Growth for Efficient Hybrid Perovskite Solar Cells. Nat. Commun..

[19] Lee M.M., Teuscher J., Miyasaka T., Murakami T.N., Snaith H.J. (2012). Efficient Hybrid Solar Cells Based on Meso-Superstructured Organometal Halide Perovskites. Science.

[20] Levchuk I., Hou Y., Gruber M., Brandl M., Herre P., Tang X., Hoegl F., Batentschuk M., Osvet A., Hock R. (2016). Deciphering the role of impurities in methylammonium iodide and their impact on the performance of perovskite solar cells. Adv. Mater. Interfaces.

[21] (2015). Extorr XT Series RGA Models: Instruction Manual.

[22] Kim B.-S., Gil-Escrig L., Sessolo M., Bolink H.J. (2020). Deposition kinetics and compositional control of vacuum-processed CH_3_NH_3_PbI_3_ perovskite. J. Phys. Chem. Lett..

[23] Gebremichael B., Alemu G., Mola G.T. (2017). Conductivity of CH_3_NH_3_PbI_3_ thin film perovskite stored in ambient atmosphere. Phys. B Condens. Matter.

[24] Zhang H., Yuan S., Qiu Z., Jiang Y., Zhu X., Wan X., Cao B. (2018). Excess Iodine As the Interface Recombination Center Limiting the Open-Circuit Voltage of CuI-Based Perovskite Planar Solar Cell. J. Mater. Sci. Mater. Electron..

[25] Kim H.D., Ohkita H., Benten H., Ito S. (2016). Photovoltaic performance of perovskite solar cells with different grain sizes. Adv. Mater..

[26] Ren X., Yang Z., Yang D., Zhang X., Cui D., Liu Y., Wei Q., Fan H., Liu S. (2016). Modulating crystal grain size and optoelectronic properties of perovskite films for solar cells by reaction temperature. Nanoscale.

[27] Nie W., Tsai H., Asadpour R., Blancon J.-C., Neukirch A.J., Gupta G., Crochet J.J., Chhowalla M., Tretiak S., Alam M.A. (2015). High-efficiency solution-processed perovskite solar cells with millimeter-scale grains. Science.

[28] Chiang C.-H., Wu C.-G. (2016). Film grain-size related long-term stability of inverted perovskite solar cells. ChemSusChem.

[29] Droseros N., Longo G., Brauer J.C., Sessolo M., Bolink H.J., Banerji N. (2018). Origin of the enhanced photoluminescence quantum yield in MAPbBr_3_ perovskite with reduced crystal size. ACS Energy Lett..

[30] Yan J., Ke X., Chen Y., Zhang A., Zhang B. (2015). Effect of modulating the molar ratio of organic to inorganic content on morphology, optical absorption and photoluminescence of perovskite CH_3_NH_3_PbBr_3_ films. Appl. Surf. Sci..

[31] Falsini N., Calisi N., Roini G., Ristori A., Biccari F., Scardi P., Barri C., Bollani M., Caporali S., Vinattieri A. (2021). Large-area nanocrystalline caesium lead chloride thin films: A focus on the exciton recombination dynamics. Nanomaterials.

