# Supplementary material for: Evaporation of Methylammonium Iodide in Thermal Deposition of MAPbI3
Source: Nanomaterials (Basel). 2021 Sep 28;11(10):2532. doi: 10.3390/nano11102532 (PMC8538304; doi:10.3390/nano11102532)
Supplement: Supplementary file 1 [file nanomaterials-11-02532-s001.zip › nanomaterials-1376216-supplementary.pdf]

nanomaterials

Supplementary Materials

Evaporation of Methylammonium Iodide in Thermal Deposition of $MAPbI_3$

Ke Wang [1], Benjamin Ecker [1], Jinsong Huang [2] and Yongli Gao [1,*]

[1] Department of Physics and Astronomy, University of Rochester, Rochester, NY 14627, USA; kwang41@ur.rochester.edu (K.W.); becker@ur.rochester.edu (B.E.)
[2] Department of Applied Physical Sciences, University of North Carolina at Chapel Hill, Chapel Hill, NC 27599, USA; jhuang@unc.edu
* Correspondence: ygao@pas.rochester.edu

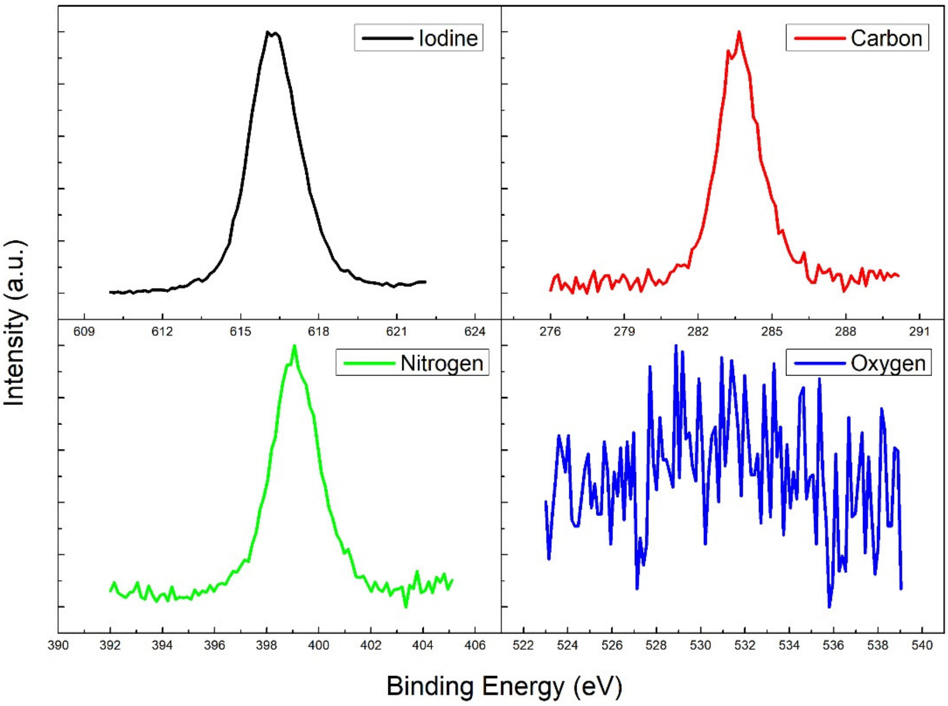

Figure S1. XPS spectra for iodine, carbon, nitrogen and oxygen of currently used MAI powder from our collabrator, respectively.

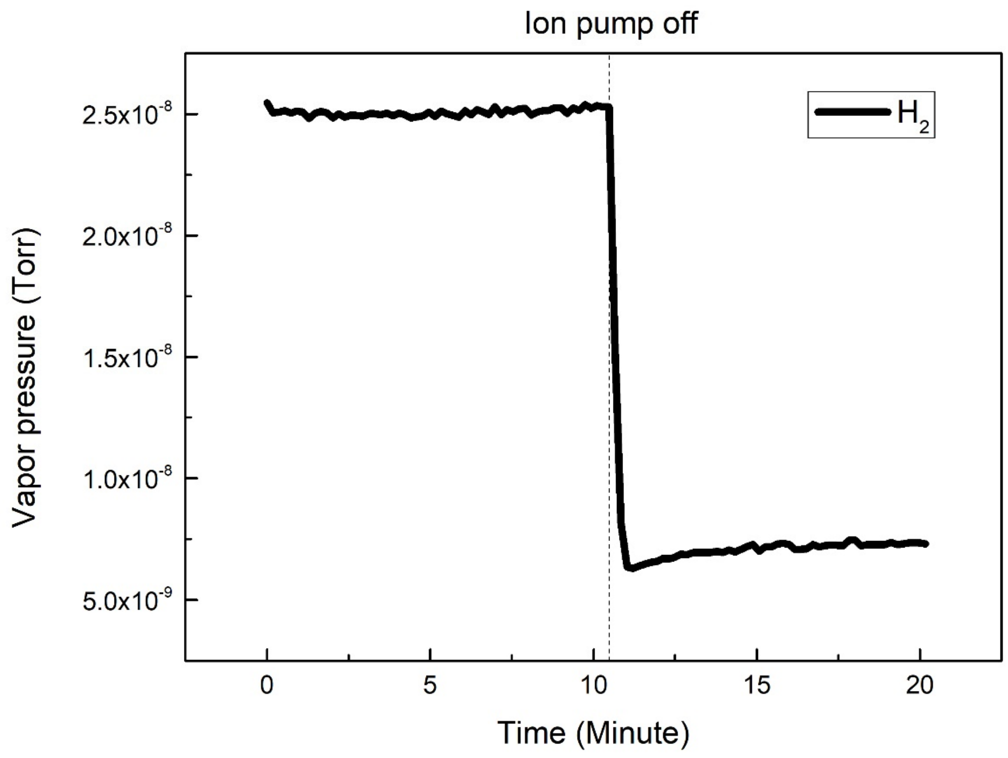

Figure S2. H_2 pressure before and after turning off ion pump.

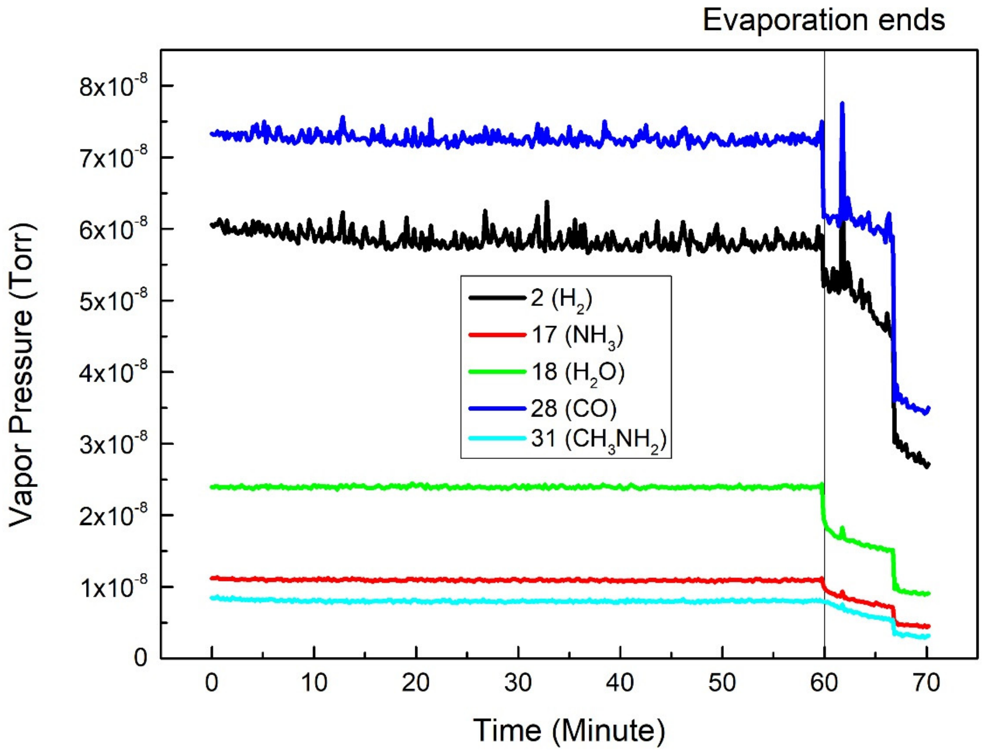

Figure S3. RGA spectra for LW/LT sample.

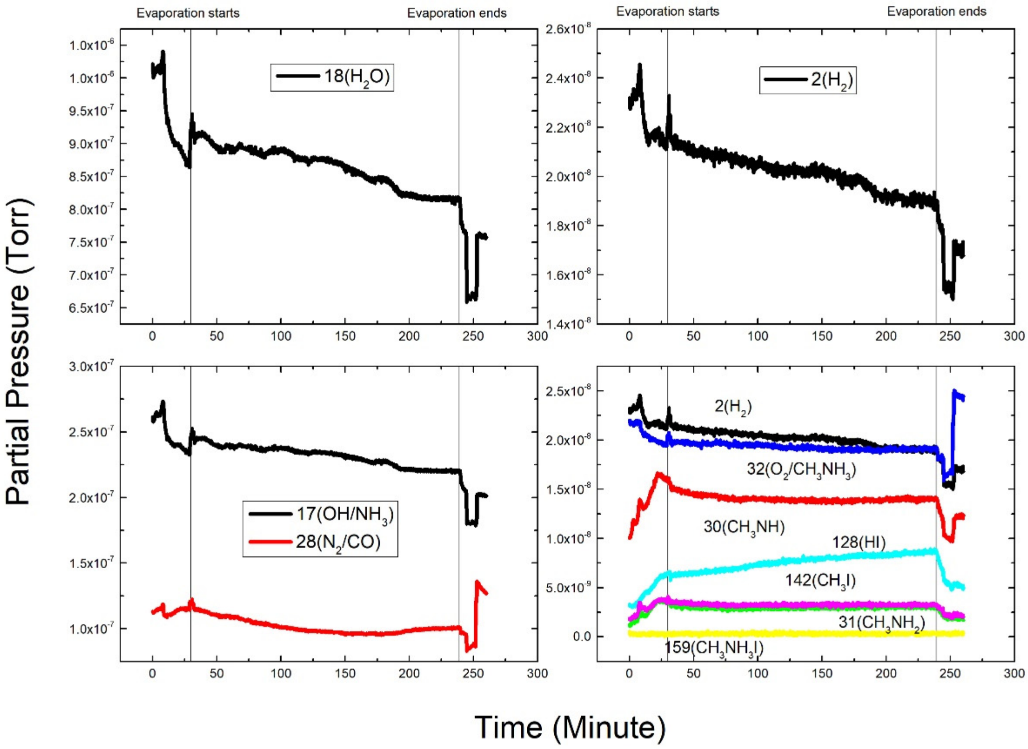

Figure S4. The RGA scans of the HW/LT evaporation. (a) AMU 18. (b) AMU 17 and 28. (c) AMU 2, 32, 30,128, 142,31 and 159. Most of the compounds went down during the evaporation since they were either participated in the reaction with water or pumped off by the turbopump. Only HI constantly went up because it did not react with water and can hardly be pumped by the turbo.

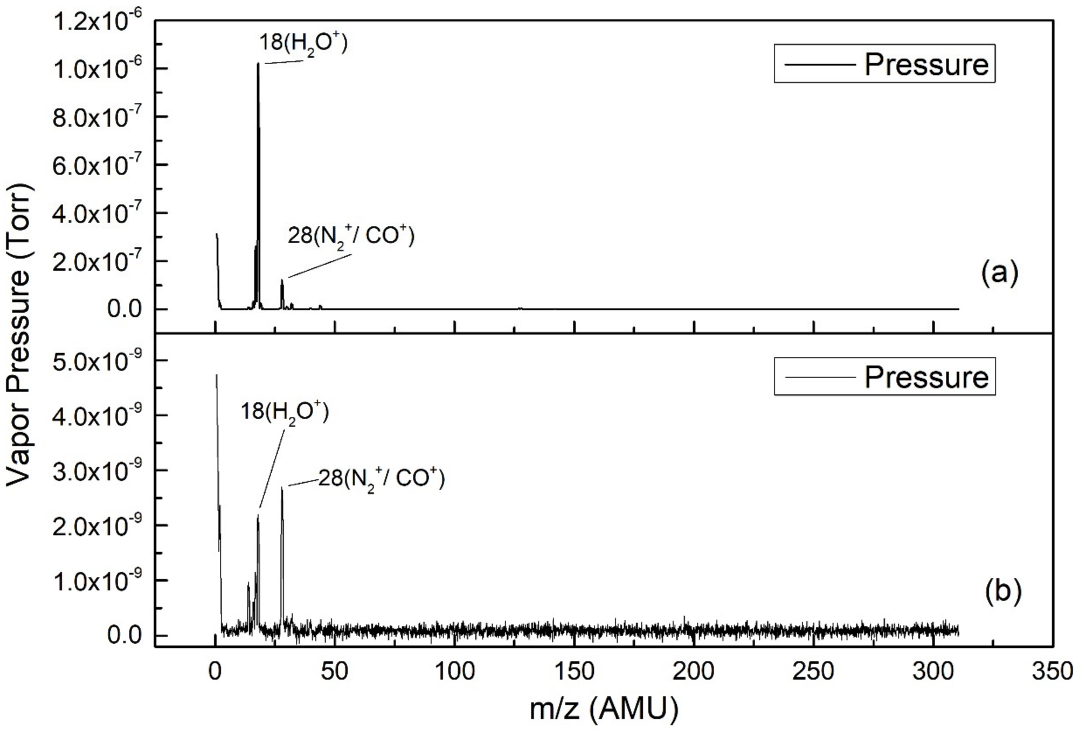

Figure S5. (**a**,**b**) RGA spectra of chamber environment before high water pressure (HW) evaporation and low water pressure (LW) evaporation, respectively.

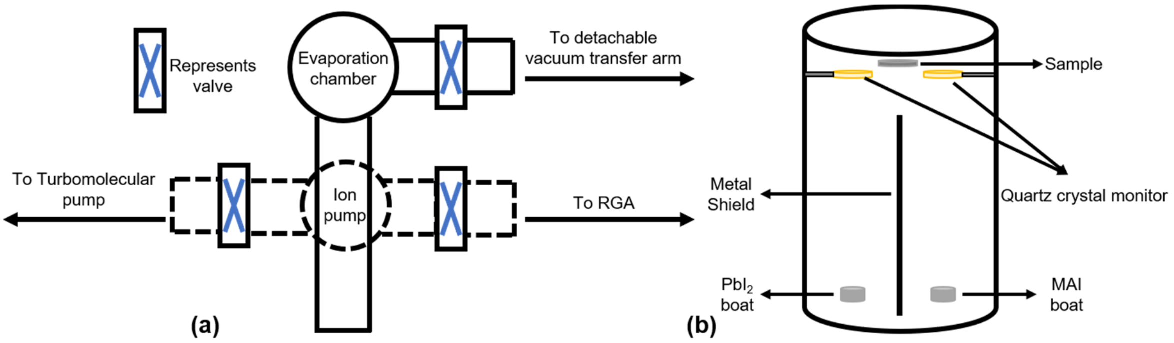

Figure S6. (**a**) Topview of evaporation chamber, the dashed components are attached to the arm from below. (**b**) Illustration of the configuration inside the evaporation chamber.

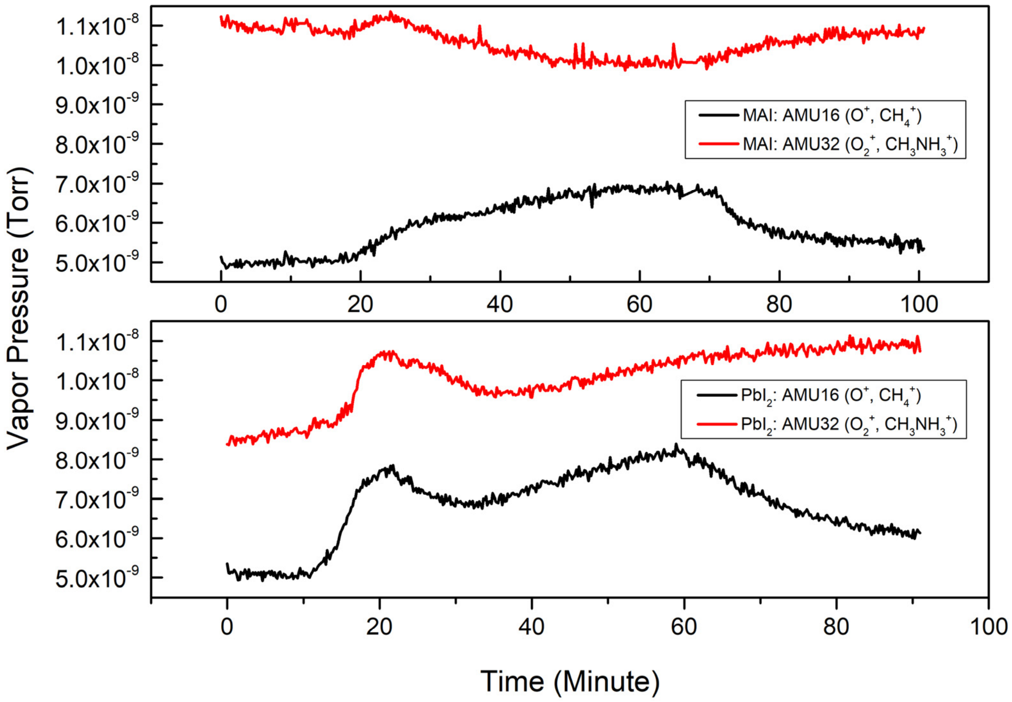

Figure S7. Comparison of AMU 16 and AMU 32 trend scans in MAI and PbI_2 evaporation. AMU 16 could be O^+ which is the O_2 fragment. AMU 32 could be O_2 or $CH_3NH_3^+$. In PbI_2 evaporation, AMU 16 showed a similar pattern to AMU 32, which indicates that AMU 32 and AMU 16 were mostly O_2 and O^+ respectively. By comparing the trends, the majority of the AMU 32 in MAI evaporation was $CH_3NH_3^+$. And it was consumed during the evaporation.

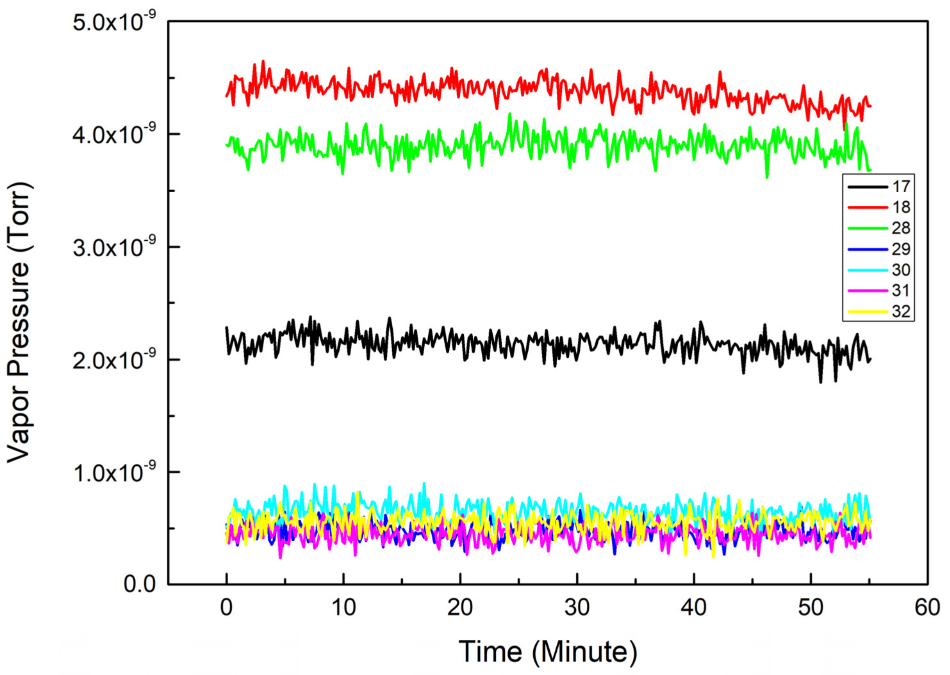

Figure S8. RGA spectra of selected compounds of an empty boat at 120 °C.

Table S1. The elemental ratio comparison of currently used MAI powder and two previous ones. Current MAI is from our collaborator. Previous #1 is from Shanghai Zhenpin limited Company. Previous #2 is from Sigma Aldrich.

Element/Ratio	Current MAI	Previous #1	Previous #2
C	0.97	1.52	1.22
N	0.87	0.94	0.87
I	1	1	1
O	0	0.15	0.19

Table S2. The elemental ratios of 10 LW/LT sample.

Element	Ratio									
I	3.42	3.06	3.09	3.31	3.11	3.02	3.18	2.83	2.93	3.03
Pb $4f_{7/2}$	1.00	1.00	1.00	1.00	1.00	1.00	1.00	1.00	1.00	1.00
Pb $4f_{5/2}$	0.77	0.76	0.77	0.77	0.77	0.75	0.79	0.76	0.79	0.78
C (286)	1.27	1.08	1.01	1.08	1.10	1.00	1.06	0.94	1.03	0.71
C (284)	0.72	0.59	0.81	0.63	0.76	0.84	0.77	0.84	0.61	0.54
N	1.10	0.77	0.70	0.91	0.85	0.78	0.88	0.47	0.79	0.51

Table S3. Comparison of AFM statistic parameters of LW and HW samples.

Statistic parameters	LW	HW
Amount of sampling	65536	65536
Sampling area	25.018 μm^2	25.000 μm^2
Mean	2.88×10^{-16} nm	-1.02×10^{-11} nm
Min	−8.243 nm	−45.606 nm
Max	10.974 nm	33.493 nm
Peak-to-peak	19.218 nm	79.099 nm
Root mean square, RMS	2.075 nm	13.781 nm
Roughness average	1.548 nm	11.428 nm
Skewness, Ssk	0.260	−0.619
Kurtosis, Ska	4.592	2.780
